# An Account of a Case of Tubercular Disease

**Published:** 1840

**Authors:** George W. Bayless

**Affiliations:** Dissector for the Pathological Cabinet of the Louisville Medical Institute


Art. VII.—*An account of a case in which there was extensive tubercular deposits in the serous membrane, sub-serous cellular tissue, and false membranes.* By George W. Bayless, M. D., Dissector for the Pathological Cabinet of the Louisville Medical Institute.

In presenting the following case for publication, I am governed rather by a desire to give as far as can be obtained, the detailed history of a patient, in which were found upon examination, some uncommon, but not new, pathological appearances, than by the belief that it will prove of much practical utility. That we are not allowed to hope for the latter, we are compelled to acknowledge, when we contemplate that the profession is so meagre in means for the prevention and cure of tubercle in any of its situations: a lamentable truth, growing out of the fact, that its nature and causes, remote and proximate, are still among the hidden things of Nature. Including the various localities which it assumes, and the various ages in which it makes its appearance, it constitutes, or more properly, lays the foundation of a large proportion of the loathsome and fatal maladies to which we are liable—presents a subject, than which there is none connected with medicine more deeply involving the best interests of humanity—affords a broad field of action for those of the profession who find an elevated pleasure in ardent, zealous labor for the furtherance of its interests—and invitingly tenders the most eminent distinction to the ambitious aspirant. But notwithstanding the great obscurity in which this matter is wrapped, when we take a retrospect of what has been accomplished in *physical* science, are we not justified in the hope, that nature will at length reveal the mystery to some one who will patiently and industriously wait upon her? And this hope is strengthened by the knowledge, that the period in the history of medicine has arrived, when instead of visionary theorizing, well authenticated facts have come to be more highly prized, and to be considered the only legitimate data upon which reasoning may be founded, and conclusions drawn. In view of this then, we conceive it to be the bounden duty of every member of the profession to make public any new or uncommon facts, which may come within his observation, especially connected with a subject of such vital consequence: a duty which he owes to his profession, and through it, to society.

The subject of this communication was a negro woman, belonging to a gentleman living in the neighborhood of this city. She was about nineteen years of age—of medium height—very spare, exhibiting a very small development of the muscular system; which appearance, we were informed, she had always presented—chest small, in both its lateral and antero-posterior diameters. She had one sister to die of phthisis, and another of an obscure disease, very similar in its symptoms, to the one with which she was affected, but in which no post-mortem examination was made. She herself had, notwithstanding her very delicate form and appearance, always enjoyed good health, until a few months after giving birth to a healthy child, in August, 1838. At the time of which we speak, she was affected with a troublesome cough, little viscid mucous expectoration, and which was attributed by her sister, to a cold which she took shortly subsequent to her delivery. It was treated during the winter with some domestic remedies, by which it was mitigated, and in the spring, being entirely relieved of it, she regained her flesh and strength, and resumed her ordinary occupation of cook. Her health continued very good, and sometime in the latter part of November, her mistress discovered that she was increasing somewhat in size. She supposed her to be pregnant, and, as the girl made no complaint, took no further notice of it. Sometime in the early part of January, perceiving that she had become very *ashy*, and that she again had a cough, similar to the one with which she had been affected the previous winter, she found upon inquiry, that the girl complained of nothing except the cough—stating, also, at the same time, that she had felt a movement, about a week before Christmas, which she supposed to be a child, and that the menses had stopped about the same time. She confessed also, that she had changed her woollen for light clothes—and had gone to a ball and danced all night, during the Christmas holidays. It had been observed also, that, about the last of December, she increased very rapidly in size; so as in a few days to present the appearance of a woman in the sixth or seventh month of utero-gestation. About the 8th or 10th of January, the time above referred to when she was first observed to look so badly, she had diminished visibly in size, but was still, from her appearance, and her own belief upon the subject, supposed to be pregnant. Some circumstances that occurred subsequent to this time, induced the suspicion that it was a case of ascites, but it was considered doubtful, and only some of the milder diuretics were employed without effect.

On the 30th of January we were called to see her, and found her in the following condition:

Some obvious emaciation; quite weak; skin generally flaccid, cool, and (upon the face) of an ashy appearance; face thin; countenance compressed and anxious; tongue very lightly covered with a white fur; and, together with the mucous membrane covering the gums and mouth generally rather pale; no thirst; appetite tolerably good, the stomach retaining very well whatever food was taken; no pain produced by pressure or percussion upon the epigastrium, or any other part of the abdomen; bowels in a very good condition; thorax, as before observed, small, but no difference in size of the two sides; did not emit, upon percussion, the resonance of a finely formed chest, with corresponding lungs, but there was no very marked flatness of sound in any part; the sub-clavicular region of each side being a little dull; the stethescope revealed only some sonorous mucous rhonchi in different portions of both sides, together with very slight blowing sound at the apex of the left lung, but quite plainly marked in the same portion of the right; cough tolerably frequent; expectoration transparent viscid mucus, with occasionally a portion of opaque mucus, such as is commonly seen in the declining stages of acute bronchitis; and, lastly, some uneasiness of respiration, but not amounting to a positive dyspnœa. There were no particular cerebral symptoms. Pulse one hundred, rather small, but somewhat tense and quick.

The next point to be ascertained was, to what must her increased size be attributed—whether pregnancy, (as she herself had believed and the family supposed,) or ascites?

In regard to the signs of pregnancy: In the first place, her size would indicate that it existed; but some circumstances connected with this appearance detracted from its importance and weight in leading to this opinion. As there was not the hard, resisting, distinct tumor, which is commonly to be felt in this state; the abdomen was very tense and dependent in the erect position, but was loose and flaccid, and had a soft doughy feel in the recumbent; the tumor assumed various positions, according to that in which the patient was placed, leaving the hypogastric and being generally diffused over the abdomen when lying on her back, and gravitating to either side as she was induced to assume a position upon one or the other; besides, there was a very distinct fluctuation discovered by percussion.

*Secondly.* There was no change in the appearance of the breasts, the nipples, aveolæ, and whole mammæ, being in a flaccid state, instead of being firm and *erect*, as would be expected at the sixth month of utero-gestation.

*Thirdly.* There was no suppression of the menses until about the time when she supposed she felt the movement of the child.

*Fourthly.* She never felt but one movement, although at least six weeks had elapsed since that time.

*Fifthly.* An examination per vaginam did not reveal any change in the neck of the uterus, and the os uteri projected into the vagina, as in the unimpregnated state, whereas we might have expected it to be almost obliterated. And again: the os uteri was down in the cavity of the pelvis, in its natural position, instead of being as high as the plane of the superior strait, as might have been expected.

*Sixthly.* No movement could be excited in the supposed existing fœtus, by the sudden application to the abdomen of the hand, which had been previously immersed in cold water.

*Seventhly.* Not the slightest pulsation of the fœtal heart could be discovered in any part of the uterine region, by means of the stethescope; neither any thing like the utero-placental soufflet.

In this detail we find only three circumstances which could lead to a suspicion of the existence of pregnancy. The tumor of the abdomen; the suppression of the menses, and the *movement* which the patient had experienced. In regard to the first, it presented none of the characters attendant upon pregnancy, while it exhibited all those of ascites; the fact of exposure to cold, above mentioned, also leading to the same conclusion. In reference to the suppression of menstruation, it could hardly be entitled to much weight in this connexion. For, although its cessation at the late period spoken of, did not preclude the possibility of utero-gestation somewhat advanced at that time, for the reason that females have been known to discharge a *sanguineous* fluid in the early months, and even up to the full term, yet the circumstance of the discharge recurring at the regular periods up to the time when she supposed that she felt the movement of the child, rendered it suspicious; and when viewed in connexion with the other circumstances of the case, together with the fact that there was entirely sufficient cause to account for the suppression, in the supposition of the existence of ascites, led to its being discarded as unworthy of any considerable weight, in determining the nature of the case. As to the *movement* spoken of, she pretended not to have felt it but the once, and we concluded that she had been mistaken in that.

These facts then, and this view of them, induced the belief that it was clearly a case of ascites: and it only remained to ascertain the cause of it. From all that we could gather, there was not the slightest reason to suppose the existence of organic disease of the kidneys, liver, spleen, or heart, and not to a sufficient degree, of the lungs, to afford any obstacle to the circulation.

We then unhesitatingly declared the opinion—that as regarded the lungs about which much anxiety had been manifested, there was some tubercles, in the first stage of their formation, in the apex of the right lung—some few, but not so many, in that of left—but there was not sufficient in quantity, nor in a state of softening, &c., to cause fear of any immediate danger from that source. Secondly, that she was not pregnant; at least, not so far advanced as her size would indicate: but that she might *possibly* be in the first or second month, on which point, we of course could not be positive. And thirdly, that it was a case of idiopathic ascites, most probably resulting from cold—and very doubtful in its issue.

With this view of the case—considering the abdomen the point of most interest and requiring immediate attention—and considering the proximate cause of the effusion to be a sub-acute inflammation of the peritoneum, which probably had not yet entirely subsided, as the pulse seemed to indicate, although there was no pain present, we could not hesitate in deciding upon the adoption of an antiphlogistic plan of treatment. We accordingly bled her to ℔ ℥x.—to aid in the diagnosis, and also to ascertain how she would bear it. The pulse became soft and feeble, and fell to about eighty towards the close of the bleeding—and she complained of feeling weak. She did not however complain but a few minutes after the arm was tied up. The blood did not exhibit any appearance of the buffy coat—there was a dark, tolerably firm clot, but very small in proportion to the quantity of serum. The pulse rose again in a few hours to one hundred, but not so tense and quick. And we will here state, that it never afterwards (until shortly before death) fell below one hundred, notwithstanding the treatment to which she was subjected: but that it never was so quick and tense as before the bleeding.

She was now put upon the following prescription:

℞ Calomel, gr. ii.

Pulv. scillœ, gr. ii.

do. digitalis, gr. i. M. ft. pulv.

The powder was given three times a day, together with a solution of sup. tart. potass. an ounce to a pint of water, to be given hourly, so that the whole should be taken in the twenty-four hours. There was generally about three dejections in twenty-four hours, thin, watery, and most commonly mixed with a light greenish bile. Urine, scanty and high colored—not coagulable by heat.

This treatment was continued four or five days, without any obvious change, when Professor Drake saw her. He concurred with us in our view of the case, and approved of the treatment in every particular. Upon finding considerable flatness of sound upon percussion, in the lower regions of the thorax when the patient was placed in the erect position, he suggested the probability of there existing an effusion in that cavity.

The treatment was continued four or five days longer, without any change in the symptoms; and the pulse continuing at one hundred or more, but not full, nor strong enough to justify the use of the lancet, we omitted the squills, thinking them too stimulating, and substituted five gr. of nit. potass. in their place—leaving off at the same time the use of the sup. tart. potass. After two or three days there was an evident increase in the quantity of urine, and seemingly a diminution in the size of the abdomen—the evacuations from the bowels being the same, and the pulse still continuing one hundred.

From this time her strength began to fail rapidly, without any change in the symptoms, save some delirium, probably produced by the digitalis, but which subsided in a few hours, upon its use being suspended for the time.

Did not see her for several days, but was told that she continued to grow rapidly weaker, and died on the 21st of February.

Examination of the corpse eighteen hours after death:

*Exterior.*—Very considerable emaciation. Abdomen very much reduced in size from what it had been, four or five days before death.

*Head*—not examined.

*Thorax.*—Upon attempting to lift up the sternum with the cartilages of the ribs, found them firmly adherent to the surface of the lungs. This adhesion existed throughout nearly the whole surface of both lungs, and so strong at the apex of the left, as to cause a considerable laceration of the substance of the lung, in the effort to detach it. There were some points of both lungs unattached, and a space of some size at the lower posterior portion of the right, in which there was about half a pint of bloody serum. There was, over the whole surface of the pleura costalis and pulmonalis, deposites of tubercular matter, about the size of common squirrel shot, only that they were compressed spheres. Some spots were larger, but none of them softened. In the apex of the right lung there were a good number of masses of tubercular matter, about the size of the extremity of the little finger, and four or five of them in that of the left lung, together with some small points throughout the substance of both lungs.

*Heart.*—In the cellular texture which lies about the pericardium, there was a considerable quantity of coagulable lymph of recent deposite. Also, one patch, nearly an inch square, on the surface of the heart, besides some smaller ones.

No lesion in the interior; but in the substance of, and beneath the lining membrane of the aorta, about half an inch from the semilunar valves, were some patches of tubercular deposite, thus showing, by analogy of lesion, the similarity of this to the serous membranes.

*Abdomen* contained between three pints and half a gallon of bloody serum.

*Stomach and Bowels*—Covering the whole of their surface, there being scarcely a naked spot as large as the extremity of the little finger, were deposites of tubercular matter, in the substance of their serous coat, and in the sub-serous cellular tissue, varying in size from that of a small shot to oval and circular patches of half an inch in diameter. The stomach was pretty firmly adherent to the concave surface of the spleen by false membrane—the bowels perfectly agglutinated into a mass, and also firmly adherent to the parietes just below the spleen. In the substance of these false membranes, particularly those uniting the convolutions of the small intestines, were masses of the same kind of matter, though not so large as some of those on the stomach and bowels.

*Mesentery.*—The same appearance was presented by its surface as by the stomach and bowels. In the cellular tissue between its laminæ, were great quantities of tubercular matter, and its ganglia were, some of them, entirely converted into the same, while others were melanotic. In the hepatico-gastric, and the gastrico-splenic omenta, were numerous ganglia, converted into tubercle—and in contact with the head of the pancreas, which, indeed, it at first seemed to be, was a mass of tubercular matter, deposited in the loose cellular tissue of this region, of an irregular cylindrical shape, about an inch in diameter and two inches in length. The great omentum was drawn up into an irregular lobulated mass, nearly an inch in thickness, and lying over the arch of the colon, in contact with the larger curvature of the stomach. It was almost a solid mass of tubercle. The peritoneum, as it covers the parietes of the abdomen—the fundus of the bladder—the uterus and ovaria, were thickly studded with this same matter, varying in size, from mere points up to that of a buck shot.

*Liver.*—This organ was paler than natural, and its whole surface covered as the stomach and bowels; the convex surface being adherent throughout to the diaphragm. There were some, but not many, small masses in its substance.

*Spleen*—united by false membrane, throughout its whole surface to the contiguous viscera; and so firmly to the diaphragm as to require a delicate dissection with the knife to detach it. When separated, its surface was found studded with small masses of tubercular matter; and when cut into, there was a considerable number of masses found in its substance, generally about the size of buck-shot. Besides these, it presented the appearance of a dark, unorganized mass, similar to a clot of very black blood.

*Uterus*—natural size of the unimpregnated state; interior surface of a bluish lead color, with two or three very small hydatids adhering to it.

*Ovaria*—natural size and appearance, save that each one contained a body about the size of a buck-shot, having very much the appearance of putty, but much harder.

It will be seen that we were correct in our diagnosis, as to the non-existence of utero-gestation, and the effusion into the cavity of the peritoneum: and, also, that it was consequent upon a chronic inflammation. But in that this inflammation was altogether attributable to *cold*, we were mistaken. Cold may, and probably did, co-operate in the production of the inflammation, with its consequences of effusion and false membrane, but that the immense deposites of tubercular matter, was the principal cause, we have no hesitation in believing. We did not suspect the existence of these deposites as they were found in the abdomen; for we knew of no sign by which they could be detected—especially when obscured by the existence of a considerable serous effusion.

Gross, in his recent work on Pathological Anatomy, under the head of tubercles of the peritoneum, says—"No particular symptoms are occasioned by the presence of these bodies; in the generality of cases they occur in scrofulous subjects, in connection with tubercles of the lungs, spleen, liver, or other organs."

The specimen was presented to the pathological cabinet of the Louisville Medical Institute, where it may be seen.

*March*, 1840.

